# Waist Circumference Might Be a Predictor of Primary Liver Cancer: A Population-Based Cohort Study

**DOI:** 10.3389/fonc.2018.00607

**Published:** 2018-12-12

**Authors:** Luopei Wei, Ni Li, Gang Wang, Xiaoshuang Feng, Zhangyan Lyu, Xin Li, Yan Wen, Yuheng Chen, Hongda Chen, Shuohua Chen, Shouling Wu, Min Dai, Jie He

**Affiliations:** ^1^Office of Cancer Screening, National Cancer Center/ National Clinical Research Center for Cancer/Cancer Hospital, Chinese Academy of Medical Sciences and Peking Union Medical College, Beijing, China; ^2^Department of Oncology, Kailuan General Hospital, Tangshan, China; ^3^Health Department of Kailuan (Group), Tangshan, China; ^4^Department of Thoracic Surgery, National Cancer Center/National Clinical Research Center for Cancer/Cancer Hospital, Chinese Academy of Medical Sciences and Peking Union Medical College, Beijing, China

**Keywords:** waist circumference, primary liver cancer, cohort studies, Chinese males, restricted cubic spline

## Abstract

**Background:** Waist circumference, as an indicator of central adiposity, has been identified as an important predictor of several specific cancers such as colorectal cancer and gastroesophageal cancer risk, however, a consensus regarding the association between waist circumference and primary liver cancer (PLC) risk has not been reached.

**Methods:** A total of 104,825 males participating in the health checkup were included in the Kailuan male cohort study (2006–2015). Information on demographic and socioeconomic characteristics, lifestyle, medical records, and anthropometric measures were collected. Restricted cubic spline (RCS) and Cox proportional hazards regression models were used to estimate the hazard ratio (HR) and 95% confidence interval (CI) of association between waist circumference and the risk of PLC in males.

**Results:** During a median of 8.9 years of follow-up, 346 PLC cases were newly diagnosed in the cohort. The RCS model showed a U-shaped association between waist circumference and PLC risk (*P-overall* = 0.019, *P-non-linear* = 0.017). Overally, males with both high waist circumference (HR_Q5vs.Q3_ = 1.98, 95%CI: 1.39–2.82) and low waist circumference (HR_Q1vs.Q3_ = 1.52, 95%CI: 1.02–2.27) had an increased risk of PLC. Especially, the U-shaped association between waist circumference and PLC risk tended to be strengthened among subjects with hepatitis B surface antigen (HBsAg) negativity (HR_Q5vs.Q3_ = 2.39, 95%*CI*: 1.43–3.98; HR_Q1vs.Q3_ = 2.27, 95%*CI* = 1.29–4.01).

**Conclusions:** Waist circumference might be an independent predictor of PLC risk in males, especially for subjects with HBsAg negativity. Controlling waist circumference in an appropriate range might be an effective primary prevention to decrease PLC risk.

## Introduction

Primary liver cancer (PLC) is one of the most common cancers. According to the estimation of GLOBOCAN 2012 by the International Agency for Research on Cancer (IARC), approximately 83% of all liver cancer occurred in less developed regions, with China accounting for over 50% of the world's burden (1).

It has been established that chronic infection with hepatitis B virus (HBV), causing chronic hepatic inflammation that may lead to fibrosis and cirrhosis, is the leading cause of PLC (2). With the successful introduction of hepatitis B vaccine into the national immunization program in China, the prevalence of hepatitis B surface antigen (HBsAg) among children under 5 years of age has dramatically declined from 9.67% in 1992 to 0.96% in 2006 (3). Hence, HBV, the dominant risk factor of PLC, is unlikely to be the main risk factor of PLC in the future. Thus, it is necessary to explore other important and potentially modifiable risk factors.

Several meta-analyses based on prospective cohort studies have identified increasing body mass index (BMI), the indicator of general adiposity which is often assumed to represent the degree of body fat, was related to higher risks of PLC (4, 5). However, abdominal fat may vary distinctly within a narrow range of BMI (6). In addition, current evidence suggests that visceral adipose is primarily found in the abdominal cavity, which had been confirmed more metabolically active than subcutaneous adipocytes (7–9). Previous study have suggested that waist circumference was a better predictor of abdominal fat compared with BMI in males (10). Hence, waist circumference, as the index considering both the amount and distribution of adipose, could be an appropriate measurement of abdominal obesity compared with BMI (11).

The recent study reported that the abdominal obesity (waist circumference ≥90 cm for male and ≥80 cm for female) prevalence was approximately quadrupled from 9.53% in 1993 to 36.7% in 2011 among Chinese males (12). Although waist circumference has been identified as an important predictor of several specific cancers such as colorectal cancer (13) and gastroesophageal cancer risk (14) in general, the association between high waist circumference and PLC risk in males has not reached a consensus (15–19). In addition, the effect of low waist circumference has rarely been investigated, leaving evidences to be further strengthened. Therefore, we conducted a large prospective cohort study based on the Kailuan Group to investigate the association between waist circumference and risk of PLC incidence in Chinese males, which might be helpful for identifying a potentially preventable risk factor of PLC.

## Methods

### Study Design and Population

The Kailuan male study, a large and dynamic prospective cohort study, was initiated in May 2006 and based on Kailuan Group in Tangshan city, Hebei province, northern China. The Kailuan Group is a functional community managing coal industry, machine manufacturing, coking, chemical engineering, transportation, new building materials, and health care institutions (including 11 affiliated hospitals) (20).

Participants were enrolled in the present study if they met the following criteria: (1) males with age>18, (2) providing informed consent, (3) completing the questionnaire interview. Participants without baseline waist circumference (*n* = 3,786), with waist circumference lower than 1st percentile (< 68 cm, *n* = 991), and with waist circumference higher than 99th percentile (>112 cm, *n* = 1,010) were excluded. Ultimately, a total of 104,825 male subjects were enrolled in the present study. This study was carried out in accordance with the recommendations of the ethical review committee of the Kailuan General Hospital. All subjects gave written informed consent in accordance with the Declaration of Helsinki.

### Exposure Assessment

Standardized questionnaire and health examination for all participants were conducted by trained doctors and nurses at baseline entry. Information on demographic and socioeconomic characteristics, lifestyle, medical records, and anthropometric measures were collected. Smoking was defined as someone has tobacco smoking at least one cigarette per week for more than 6 consecutive months and was categorize as “non-smoker,” “ex-smoker,” or “current smoker” according to questionnaire information. Alcohol drinking was defined as drinking at least once per month for more than 6 consecutive months and was classified into “non-drinker,” “ex-drinker,” “<1 time per day” or “≥1 time per day” using self-reported information. The subjects' weights and heights were measured using standardized stadiometers and scales while wearing light clothes, and the BMI was calculated based on the formula that BMI = weight (kg)/height^2^ (m^2^). Waist circumference was measured at the midpoint between the lower border of the rib and the supra margin of iliac crest plane. Diabetes history was categorized as “yes” or “no” on the basis of fasting blood glucose (FBG) level according to diabetes diagnostic criteria recommended by International Diabetes Federation (FBG ≥ 7.0 mmol/L) (21) and history for antidiabetic medication use. Measurement of FBG was performed using the Hexokinase method (BioSino Bio-Technology & Science Inc., China.). The HBsAg was detected quantitatively by the enzyme-linked immunosorbent assay for HBsAg (SHANGHAI KEHUA BIO-ENGINEERING, KHB, Shanghai, China) with standard operating procedure.

### Outcome Assessment

The follow-up of each participant terminated at diagnosis of cancer, death, or administrative censoring (December 31, 2015), whichever occurred first. During the study period, new cases were obtained through self-report when they took part in routine questionnaires and health examinations every 2 years until 31 December 2015. In addition, incident PLC cases were checked yearly by the diagnosis and medical records linkage with the Tangshan medical insurance system and Kailuan social security system. Moreover, discharge lists from the 11 affiliated hospitals and death certificates from state vital statistics offices were also tracked yearly to ascertain the outcome information (22).

The diagnosis of incident PLC cases was confirmed by medical records review by clinical experts. Information on pathological diagnosis, imaging diagnoses (including ultrasonography, computerized tomography, and magnetic resonance imaging), blood biochemical and alpha fetoprotein test was collected to assess the incident PLC cases (22). All PLC events were coded as C22 according to the International Classification of Diseases, Tenth Revision (ICD−10). Other details relating to Kailuan Cohort has been described previously (22–24).

### Statistical Analyses

Subjects were grouped into quintiles according to the baseline waist circumference (< 80.0, 80.0–84.9, 85.0–89.9, 90.0–94.9, or ≥95.0 cm), and the third quintile of waist circumference (85.0–89.9 cm) served as the reference. Proportions and chi-square tests were used to describe the categorical variables. A restricted cubic spline (RCS) analysis was conducted to explore the potential non-linear relationship between continuous waist circumference and the risk of PLC in the study (25).

Furthermore, Cox's proportional hazards regression models were constructed to estimate the hazard ratio (HR) and 95% confidence interval (CI) of PLC risk according to waist circumference quintiles. In model 1, only waist circumference was included in this univariate model. In model 2, age (continuous) was added as the underlying time metric. In model 3, multiple factors including education level (illiterate/primary school, junior high school, senior high school, or college and above), dust exposure (no or yes), frequency of alcohol drinking (non-drinker, ex-drinker, < 1 time per day, or ≥1 time per day), and smoking status (non-smoker, ex-smoker, or current smoker) were further adjusted. In model 4, disease history including diabetes (yes or no), and HBV infection status (HBsAg negative or positive) served as additional adjustments. In model 5, the main model, BMI (continuous) was added in this multivariate model for exploring whether waist circumference is independent of BMI for PLC prediction.

Subgroup analyses were performed by alcohol drinking status (non-drinker vs. drinker), smoking status (non-smoker vs. smoker), and HBsAg status (negative vs. positive). And the multiplicative models were applied to test for the interaction between waist circumference and these variables.

Sensitivity analyses were conducted to examine the consistency of our findings. Firstly, the PLC cases occurred in the initial 3 years of follow-up were excluded from the analyses to evaluate whether potential preexisting disease influenced the association between waist circumference and PLC risk. Secondly, main models were repeated with exclusion of subjects with BMI < 18.5 kg/m^2^ in consideration of the effect of preclinical cancers that may cause weight loss and waist circumference decrement and thus result in overestimation of the association between lower waist circumference and PLC risk.

The data management and all analyses were conducted using SAS 9.4 (SAS Institute Inc., Cary, NC, USA). All statistical test presented were two-side, and *P* < 0.05 was considered statistically significant.

## Results

### Baseline Participant Characteristics

A total of 104,825 males were included in this study with a mean age of 51.4 years, for a total of 827,352.43 person-years. During a median follow-up time of 8.9 years, 346 members of the cohort were diagnosed with PLC. We compared the characteristics at baseline according to waist circumference quintiles of all subjects. As shown in Table 1, compared with subjects with low waist circumference, those with higher waist circumference were more likely to be older and have lower education level. Males in the higher waist circumference categories were more likely to be non-smokers and ex-drinkers. Negative HBsAg and diabetes were more common among males with higher waist circumference (Table 1).

**Table 1:** Baseline characteristics of males stratified by waist circumference, Kailuan male cohort, 2006–2015.

**Characteristics**	**Total No. (%)**	**Waist circumference (cm)**					**χ^**2**^**	***P*-value**
		**< 80.0**	**80.0–84.9**	**85.0–89.9**	**90.0–94.9**	**≥95.0**		
**No. study participants**	104,825 (100.00)	16,269 (15.52)	20,898 (19.94)	23,452 (22.37)	20,061 (19.14)	24,145 (23.03)		
**Age (year)**							2,230.86	< 0.001
< 40	18,849 (17.98)	4,711 (28.96)	4,132 (19.77)	4,138 (17.64)	2,915 (14.53)	2,953 (12.23)		
40~	23,099 (22.04)	3,353 (20.61)	4,879 (23.35)	5,578 (23.78)	4,453 (22.2)	4,836 (20.03)		
50~	36,792 (35.10)	4,868 (29.92)	7,315 (35.00)	8,363 (35.66)	7,447 (37.12)	8,799 (36.44)		
60~	26,085 (24.88)	3,337 (20.51)	4,572 (21.88)	5,373 (22.91)	5,246 (26.15)	7,557 (31.30)		
**Education**							256.86	< 0.001
Illiteracy/primary school	12,011 (11.51)	1,833 (10.64)	2,216 (11.30)	2,498 (10.69)	2,358 (11.83)	3,106 (12.96)		
Junior high school	69,474 (66.60)	9,913 (67.92)	14,143 (61.13)	16,000 (68.45)	13,307 (66.75)	16,111 (67.21)		
Senior high school	14,388 (13.79)	2,871 (13.45)	2,800 (17.70)	3,110 (13.31)	2,660 (13.34)	2,947 (12.29)		
College or above	8,447 (8.10)	1,599 (7.99)	1,664 (9.86)	1,766 (7.56)	1,612 (8.09)	1,806 (7.53)		
**Dust exposure**								
No	41959 (40.25)	6045 (37.32)	8442 (40.59)	9468 (40.53)	7797 (39.13)	10207 (42.62)	66.69	< 0.001
Yes	62275 (59.75)	10153 (62.68)	12356 (59.41)	13892 (59.47)	12130 (60.87)	13744 (57.38)		
**Drinking**							191.47	< 0.001
Non-drinker	52,360 (50.05)	7,914 (48.70)	10,439 (50.02)	11,953 (51.06)	9,585 (47.90)	12,469 (51.77)		
Ex-drinker	4,528 (4.33)	567 (3.49)	780 (3.74)	926 (3.96)	984 (4.92)	1,271 (5.28)		
< 1 time per day	25,873 (24.73)	4,532 (27.89)	5,154 (24.7)	5,669 (24.22)	5,084 (25.41)	5,434 (22.56)		
≥1 time per day	21,862 (20.90)	3,236 (19.92)	4,495 (21.54)	4,862 (20.77)	4,357 (21.77)	4,912 (20.39)		
**Smoking**							314.72	< 0.001
Non-smoker	54,665 (56.59)	8,037 (56.29)	10,941 (52.79)	12,599 (57.86)	10,292 (56.05)	12,796 (58.69)		
Ex-smoker	3,995 (4.14)	505 (3.19)	621 (3.32)	856 (3.93)	847 (4.61)	1,166 (5.35)		
Current smoker	37,943 (39.28)	6,682 (40.52)	7,876 (43.89)	8,321 (38.21)	7,223 (39.34)	7,841 (35.96)		
**BMI(kg/m**^**2**^**)**							26,171.84	< 0.001
< 18.5	2,282 (2.18)	1,265 (7.78)	442 (2.12)	263 (1.12)	133 (0.66)	179 (0.74)		
18.5~	61,675 (58.87)	14,094 (86.66)	16,796 (80.42)	14,997 (63.99)	9,153 (45.66)	6,635 (27.50)		
25.0~	36,399 (34.75)	850 (5.23)	3,546 (16.98)	7,885 (33.64)	10,281 (51.29)	13,837 (57.35)		
30.0~	4,404 (4.20)	55 (0.34)	102 (0.49)	293 (1.25)	477 (2.38)	3,477 (14.41)		
**HBsAg status**							20.59	< 0.001
Negative	96,750 (96.65)	15,046 (96.31)	19,234 (96.29)	21,760 (96.76)	18,456 (96.8)	22,254 (96.98)		
Positive	3,353 (3.35)	577 (3.69)	742 (3.71)	729 (3.24)	611 (3.2)	694 (3.02)		
**Diabetes history**							1212.49	< 0.001
No	92052 (90.37)	15241 (95.13)	19026 (92.85)	20835 (90.96)	17153 (88.74)	19797 (85.64)		
Yes	3319 (9.63)	780 (4.87)	1466 (7.15)	2071 (9.04)	2176 (11.26)	3319 (14.36)		

*BMI, body mass index; HBsAg, hepatitis B surface antigen*.

### The Association Between Waist Circumference and PLC Risk

**Figure 1:**
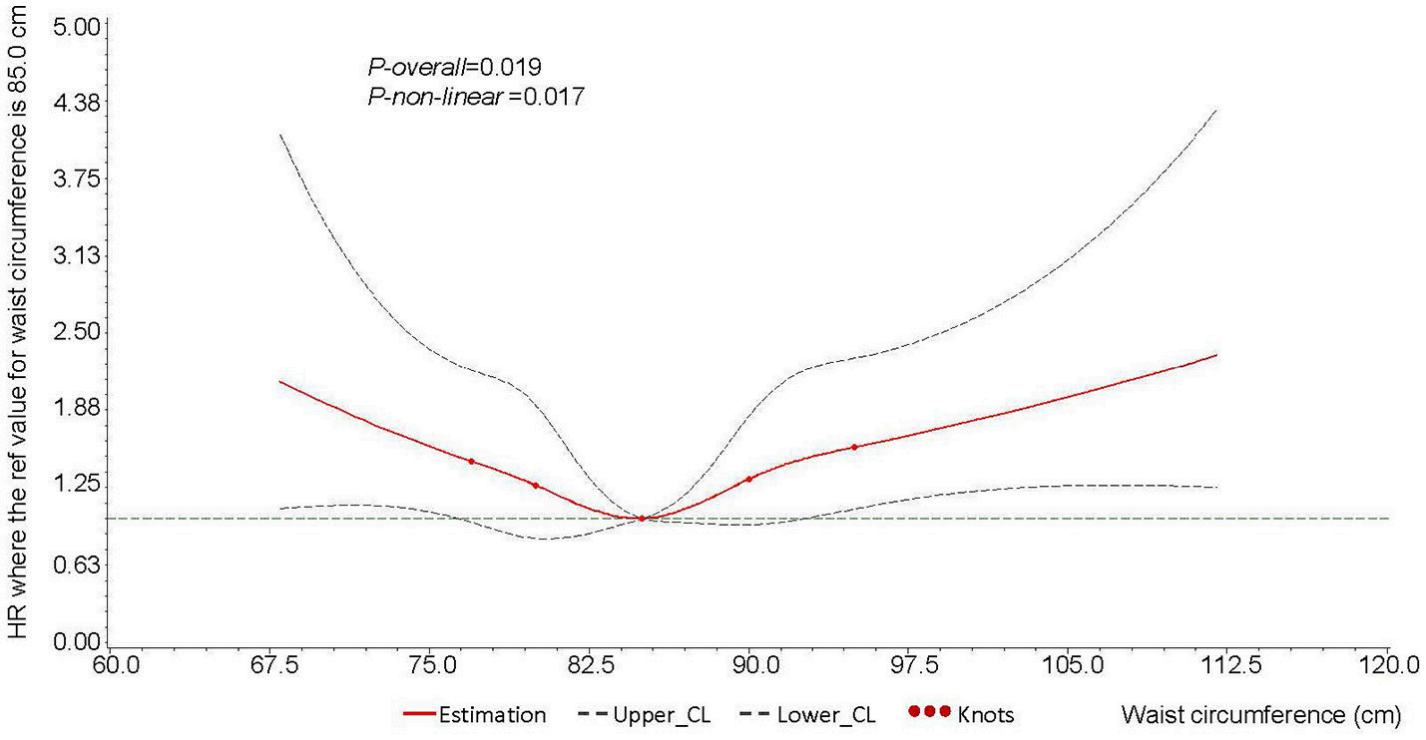
Cubic spline graph of the adjusted HR (represented by solid line) and 95%*CI* (represented by the dotted lines) for the association between waist circumference and risk of male liver cancer in Kailuan male cohort, 2006–2015. **Knots:** 77.0, 80.0, 85.0, 90.0, 95.0 of the distribution of waist circumference (cm). **Referent**: 85.0 cm, 40th of the distribution of waist circumference. Adjusted for age (continuous), education level (illiteracy/primary school, junior high school, senior high school, or college and above), dust exposure (no or yes), smoking (non-smoker, ex-smoker, or current smoker), drinking (non-drinker, ex-drinker, < 1 time per day, or ≥1 time per day), diabetes (no or yes), HBsAg (negative or positive), and BMI (continuous). HR, hazard ratio; HBsAg, hepatitis B surface antigen; BMI, body mass index.

The RCS model showed a significantly U-shaped association of waist circumference with the risk of PLC among the participants (*P-overall* = 0.019, *P-non-linear* = 0.017) (Figure 1). As the 40th quintile of waist circumference (85.0 cm) was chosen to be the reference, the HRs of PLC related to waist circumference rise obviously when waist circumference was over 95.0 cm or lower than 75.0 cm.

Furthermore, subjects were grouped into quintiles according to the baseline waist circumference, the crude PLC incidence rates for males according to waist circumference quintiles were 44.93/10^5^, 35.26/10^5^, 32.25/10^5^, 35.94/10^5^, and 59.75/10^5^, respectively. Compared with the third quintile waist circumference (85.0–89.9 cm), the HRs were 1.98 (95% CI: 1.39–2.82) for highest quintile waist circumference (≥95.0 cm) and 1.52 (95% CI: 1.02–2.27) for lowest quintile waist circumference (< 80.0 cm), respectively, after adjusting for age, education, dust exposure, status of tobacco smoking and alcohol drinking, diabetes history, HBsAg status and BMI (Table 2).

**Table 2:** The association between waist circumference and primary liver cancer in males, Kailuan male cohort, 2006–2015.

**Model**	**Waist circumference(cm)**				
	**<80.0**	**80.0–84.9**	**85.0–89.9**	**90.0–94.9**	**≥95.0**
Person-years (Case No.)	129,096.88 (58)	164,484.92 (58)	186,046.82 (60)	158,615.61 (57)	189,108.2 (113)
Incidence(1/10^5^)	44.93	35.26	32.25	35.94	59.75
Model 1 [HR (95% CI)]	1.39 (0.97–2.00)	1.10 (0.76–1.57)	Ref	1.12 (0.78–1.60)	1.86 (1.36–2.54)
Model 2 [HR (95% CI)]	1.50 (1.04–2.15)	1.12 (0.78–1.60)	Ref	1.07 (0.74–1.54)	1.69 (1.24–2.32)
Model 3 [HR (95% CI)]	1.60 (1.10–2.33)	1.23 (0.84–1.78)	Ref	1.12 (0.77–1.65)	1.90 (1.37–2.65)
Model 4 [HR (95% CI)]	1.54 (1.05–2.27)	1.11 (0.75–1.63)	Ref	1.16 (0.79–1.71)	1.96 (1.40–2.74)
Model 5 [HR (95% CI)]	1.52 (1.02–2.27)	1.10 (0.75–1.62)	Ref	1.16 (0.79–1.72)	1.98 (1.39–2.82)

*HR, hazard ratio; CI, confidence intervals; HBsAg, hepatitis B surface antigen; BMI, body mass index*.

*Model 1: Univariate model including waist circumference (< 80.0, 80.0–84.9, 85.0–89.9, 90.0–94.9, or ≥95.0 cm)*.

*Model 2: Model 1+age (continuous)*.

*Model 3: Model 2+education level (illiteracy/primary school, junior high school, senior high school, or college and above)+ dust exposure (no or yes)+smoking(non-smoker, ex-smoker, or current smoker)+alcohol drinking(non-drinker, ex-drinker, < 1 time per day, or ≥ 1 time per day)*.

*Model 4: Model 3+ diabetes (no or yes) + HBsAg (negative or positive)*.

*Model 5: Model 4+BMI (continuous)*.

Population attributable fractions (PAFs) for categorical exposure variables were calculated to reveal the common risk factors' contribution to PLC incidence. As shown in the Supplementary Table S1, in addition to HBsAg status (45.64%), the waist circumference (23.01%) also account the main attributable proportions of PLC incidence (Supplementary Table S1 in Supplementary Material).

### Subgroup Analyses Between the Waist Circumference and PLC Risk

Subgroup analyses showed that the statistically significant U-shaped association between waist circumference and PLC risk tended to be strengthened among subjects with hepatitis B surface antigen (HBsAg) negativity (HR_Q5vs.Q3_ = 2.39, 95%*CI*: 1.43–3.98; HR_Q1vs.Q3_ = 2.27, 95%*CI* = 1.29–4.01). In addition, high waist circumference (≥95.0 cm) among non-drinkers (HR = 2.14, 95%CI = 1.34–3.41) and non-smokers (HR = 1.80, 95%CI = 1.13–2.86) also indicated a positive association with PLC risk in present study. Interaction analyses were conducted between the waist circumference and these confounders. However, there was no evidence of interaction effect (all *P* > 0.05) between waist circumference and alcohol drinking, tobacco smoking and HBsAg status (Table 3).

**Table 3:** Stratified analysis of the association between waist circumference and risk of primary liver cancer in Kailuan male cohort, 2006–2015.

	**Waist circumference (cm)**					***P_***interaction***_***
	**<80.0**	**80.0–84.9**	**85.0–89.9**	**90.0–94.9**	**≥95.0**	
**Drinking**						0.195
**Non-drinker**						
Person-years (Case No.)	61,374.38 (31)	80,863.64 (23)	93,712.25 (31)	74,794.84 (26)	96,473.2 (66)	
HR (95% CI)^a^	1.68 (1.00–2.82)	0.77 (0.43–1.37)	Ref	1.00 (0.57–1.75)	2.05 (1.31–3.22)	
HR (95% CI)^b^	1.59 (0.93–2.74)	0.75 (0.42–1.35)	Ref	1.02 (0.58–1.78)	2.14 (1.34–3.41)	
**Drinker^c^**						
Person-years (Case No.)	67,612.11 (27)	83,435.57 (35)	92,094.97 (28)	83,510.19 (30)	92,265.82 (46)	
HR (95% CI)^a^	1.47 (0.83–2.59)	1.48 (0.87–2.52)	Ref	1.34 (0.78–2.33)	1.83 (1.10–3.04)	
HR (95% CI)^b^	1.56 (0.86–2.83)	1.52 (0.89–2.60)	Ref	1.30 (0.75–2.27)	1.70 (0.98–2.95)	
**Smoking**						0.809
**Non-smoker**						
Person-years (Case No.)	63,241.66 (28)	85,686.21 (27)	99,570.37 (32)	81,159.41 (27)	100,186.22 (56)	
HR (95% CI)^a^	1.49 (0.89–2.52)	0.89 (0.52–1.54)	Ref	1.09 (0.64–1.84)	1.71 (1.09–2.68)	
HR (95% CI)^b^	1.40 (0.82–2.41)	0.87 (0.50–1.50)	Ref	1.11 (0.65–1.88)	1.80 (1.13–2.86)	
**Smoker^d^**						
Person-years (Case No.)	57,893.73 (28)	67,854.07 (30)	73,581.92 (22)	64,563.19 (25)	70,931.96 (52)	
HR (95% CI)^a^	1.67 (0.94–2.95)	1.38 (0.79–2.43)	Ref	1.27 (0.71–2.27)	2.24 (1.34–3.74)	
HR (95% CI)^b^	1.76 (0.97–3.21)	1.41 (0.80–2.49)	Ref	1.23 (0.69–2.22)	2.09 (1.20–3.64)	
**HBsAg status**						0.192
**Negative**						
Person-years (Case No.)	121,737.26 (33)	155,114.28 (23)	176,440.05 (25)	149,666.76 (34)	178,641.71 (65)	
HR (95% CI)^a^	2.18 (1.26–3.76)	1.10 (0.60–2.00)	Ref	1.61 (0.93–2.78)	2.49 (1.52–4.06)	
HR (95% CI)^b^	2.27 (1.29–4.01)	1.12 (0.61–2.04)	Ref	1.58 (0.91–2.74)	2.39 (1.43–3.98)	
**Positive**						
Person-years (Case No.)	4,653.08 (24)	5,921.55 (34)	5,900.87 (35)	4,904.05 (22)	5,420.1 (46)	
HR (95% CI)^a^	1.07 (0.62–1.86)	1.09 (0.65–1.81)	Ref	0.83 (0.47–1.46)	1.56 (0.97–2.50)	
HR (95% CI)^b^	0.99 (0.56–1.76)	1.05 (0.63–1.75)	Ref	0.85 (0.48–1.52)	1.68 (1.02–2.77)	

*HR, hazard ratio; CI, confidence intervals; HBsAg, hepatitis B surface antigen; BMI, body mass index*.

a *Adjust for age (continuous), education level (illiteracy/primary school, junior high school, senior high school, or college and above), dust exposure (no or yes), smoking (non-smoker, ex-smoker, or current smoker), alcohol drinking (non-drinker, ex-drinker, < 1 time per day, or ≥1 time per day), diabetes (no or yes), HBsAg (negative or positive)*.

b *Further adjust for BMI (continuous)*.

c *Including ex-drinkers and current drinkers*.

d *Including ex-smokers and current smokers*.

### Sensitivity Analysis

As shown in Table 4, after excluding PLC cases (case No. = 127) having occurred during the first 3 years of follow-up, there was still a positive association of the risk of PLC related to high waist circumference (HR = 1.78, 95% CI: 1.18–2.69). When excluding individuals without or with BMI < 18.5 kg/m^2^ (*n* = 2,347, case No. = 11), the results did not change substantially (HR_Q5vs.Q3_ = 1.90, 95%*CI*: 1.32–2.72; HR_Q1vs.Q3_ = 1.51, 95%*CI* = 1.00–2.28).

**Table 4:** Sensitivity analysis of the association between waist circumference and primary liver cancer risk in Kailuan male cohort, 2006–2015.

	**Waist circumference (cm)**				
	**<80.0**	**80.0–84.9**	**85.0–89.9**	**90.0–94.9**	**≥95.0**
**Exclude cases occurred in the first 3 years of follow-up**					
Person-years (Case No.)	129,075.93 (39)	164,437.11 (30)	186,017.32 (45)	158,579.5 (32)	189,053.03 (73)
HR (95% CI)^a^	1.29 (0.82–2.03)	0.75 (0.46–1.21)	Ref	0.84 (0.52–1.34)	1.61 (1.09–2.38)
HR (95% CI)^b^	1.16 (0.72–1.85)	0.72 (0.44–1.16)	Ref	0.87 (0.54–1.41)	1.78 (1.18–2.69)
**Exclude BMI < 18.5 kg/m**^**2**^					
Person-years (Case No.)	119,619.69 (52)	161,043.56 (57)	183,970.46 (59)	157,445.81 (57)	187,580.26 (110)
HR (95% CI)^a^	1.49 (1.00–2.21)	1.12 (0.76–1.66)	Ref	1.17 (0.79–1.73)	1.93 (1.37–2.71)
HR (95% CI)^b^	1.51 (1.00–2.28)	1.13 (0.76–1.67)	Ref	1.17 (0.79–1.73)	1.90 (1.32–2.72)

*BMI, body mass index; HR, hazard ratio; CI, confidence intervals; HBsAg, hepatitis B surface antigen*.

a *Adjust for age (continuous), education level (illiteracy/primary school, junior high school, senior high school, or college and above), dust exposure (no or yes), smoking (non-smoker, ex-smoker, or current smoker), alcohol drinking (non-drinker, ex-drinker, < 1 time per day, or ≥ 1 time per day), diabetes (no or yes), HBsAg (negative or positive)*.

b *Further adjust for BMI (continuous)*.

## Discussion

In this large prospective cohort study among Chinese males, we found a significant U-shaped association between waist circumference and PLC risk. The association was robust even after including BMI in the statistical models, supporting the hypothesis that waist circumference is an independent predictor for PLC. In addition, the subgroup analyses showed that the association between waist circumference and risk of PLC differed across categories of alcohol drinking, tobacco smoking, and status of HBV infection, as there were discrepancies among subgroups. To our knowledge, this is the first prospective cohort study to report on the association of both high and low waist circumference with PLC risk in mainland Chinese males which could be a strong evidence suggesting waist circumference is an independent predictor of PLC.

Waist circumference was one of the earliest means of quantifying body fat distribution, as an approximation of central adiposity (26). Results from several prospective cohort studies have examined the association between high waist circumference and risk of PLC (15–19). European Prospective Investigation into Cancer and Nutrition study identified 177 liver cancer cases and reported that high waist circumference was related to higher risk of liver cancer (highest tertile VS. lowest tertile, HR = 2.60, 95% CI: 1.66–4.07) (17). Similarly, the Liver Cancer Pooling Project also found waist circumference to be an independent risk factor for liver cancer risk in males (waist circumference ≥110 cm VS. < 90 cm, HR = 1.88, 95% CI: 1.42–2.49) (15). A study from Taiwan reported that the association between central obesity (waist circumference >90 cm for men and < 80 cm for women) and PLC was only restricted in subjects with HBsAg negative and antibody to hepatitis C virus (HCV) positive (HR = 2.16, 95% CI: 1.19–3.92) (18). Our results on waist circumference were in line with the previous findings, whereby we consistently observed a significant association between high waist circumference and high liver cancer risk, even after further adjustment for BMI.

However, few studies have explored the association between low waist circumference and risk of PLC. Our study added a new perspective that the statistically U-shaped association between waist circumference and PLC risk, in which that low waist circumference might also play a potential role in PLC incidence. The RCS model showed that PLC risk increased obviously when waist circumference was lower than 75.0 cm. In addition, the present study also found a significant relationship when the first quintile (< 80.0 cm) compared to third quintile (85.0–89.9 cm) in subjects with HBsAg negativity, which support an association between low waist circumference and PLC risk. Perhaps males with low waist circumference were prone to accompany with preclinical disease that can cause weight loss and also increase risk of PLC, which may confuse the association. However, the association between low waist (< 80.0 cm) circumference and PLC risk remained robust with exclusion of participants with BMI < 18.5 kg/m^2^ (HR = 1.51, 95% CI: 1.00–2.28). In addition, for subjects with HBsAg negativity, the association was stronger (HR = 2.29, 95% CI: 1.28–4.08, data was not shown) when excluded the underweight participants (BMI < 18.5 kg/m^2^). Therefore, the robust findings indicated that low waist circumference might be a risk factor of PLC.

The previous study suggested that waist circumference was a better predictor of abdominal adiposity in males when compared with body weight or BMI (11). In our study, waist circumference conveyed statistically significant association with PLC risk, even after adjusting BMI in the statistical models, supporting the hypothesis that waist circumference is an independent predictor for PLC. Subjects with high waist circumference tend to be diagnosed with PLC maybe due to the following possible mechanisms. Increased release from metabolically active abdominal fat of substantial adipokines, such as tumor-necrosis factor-α, free fatty acids, leptin and inflammatory markers, and reduced release of adiponectin, contribute to development of insulin resistance, compensatory and chronic hyperinsulinaemia (8, 9, 26–28). Increased insulin levels, in turn, lead to reduced insulin-like growth factor (IGF) binding protein 1 synthesis in liver and other tissues, additionally, generally accompany with reduced levels of IGF binding protein 2 in the blood. Both the reduced IGF binding protein 1 and IGF binding protein 2 give rise to facilitate the biological activity of IGF1. Ultimately, insulin and IGF1 signal through the insulin receptors and IGF1 receptor, respectively, to promote cellular proliferation, inhibit apoptosis, and then contribute to tumorigenesis (8, 17). The mechanisms for subjects with low waist circumference also related to high PLC risk is still inconclusive, hence further research to better understand the underlying mechanisms are needed.

In the present study, the association between high waist circumference and risk of PLC differed by status of drinking and smoking. The association was statistically significant in non-drinkers or non-smokers but negative in drinkers or smokers. It is possible owing to the competing risks of tobacco smoking and alcohol drinking. Previous studies have suggested that alcohol drinking may increase 179% (*95%CI*: 2.00-3.87) risk of liver cancer incidence (29) via the induction of cytochrome P-450 2E1, which potentially leads to activation of procarcinogen (30) and inhibition of phase II enzymes (31), thus affecting the clearance of carcinogens (32). And tobacco smoking (HR = 1.51, 95% CI: 1.37–1.67) was also found to be an independent risk factor for liver cancer (33). Therefore, in the presence of a competing risk, the association may be attenuated among drinkers and smokers. However, for non-drinkers, or non-smokers, high waist circumference showed a significant effect on PLC development which could have key scientific and clinical importance for preventing PLC.

The prevalence of HBsAg was 3.30% in the present study, which was similar to the previous study on HBsAg prevalence (< 4%) among northern Chinese population (34). As it was estimated that hepatitis B viral infections accounts for more than 60% of liver cancer cases in Asia area (2), our study also proved that HBsAg positivity increased 30.74 (*95%CI*: 24.51–38.55) fold higher risk of PLC when compared with HBsAg negative. Therefore, the effect of waist circumference may be weakened, which explain the finding that increased PLC risk related to higher and lower waist circumference was restricted in subjects with HBsAg negativity. Although the HBV infection currently plays a leading role in the development of PLC (35), it is unlikely to be the main risk factor in the future, as the prevalence of HBsAg among the children under 5 years of age decreasing from 9.67% in 1992 to 0.96% in 2006 (3) with the successful massive hepatitis B vaccination implementation. Whereas, the abdominal obesity prevalence was approximately quadrupled from 9.53% in 1993 to 36.7% in 2011 among Chinese males (13). Thus, the findings of abdominal obesity, related to PLC risk differently depending on HBsAg status may shed some light on preventing PLC in the condition of hepatitis B vaccination application.

One of the main strengths of our study is its prospective design and inclusion of a large population, which gave us high power to detect quite modest associations as well as minimize the potential bias caused by preclinical disease. Furthermore, in our study, anthropometric factors (e.g., waist circumference, weight, and height) at baseline were measured by trained personnel rather than relied on self-reported, which avoid misclassification in analyses. However, there are several limitations that should be discussed when interpreting the results. Firstly, the lack of information on HCV infection is a major limitation of the current study. However, the HCV prevalence rate was only 0.43% in Chinese general population according to a national survey carried in 2006 (36), which attribute little to PLC incidence in China. In subsequent questionnaire interview and health examination, we will complement the HCV infection information to provide more comprehensive results in the future. Secondly, the follow-up time (Median, 8.9 years) was relatively short, which precluded stratified analyses by subtypes of PLC, such as hepatocellular carcinoma and intrahepatic cholangiocarcinoma, owing to limited number of cases. In addition, the subjects focused on male employees from Kailuan Group, it may be difficult in extrapolating to females or general population. So for other population, more studies are warranted to confirm these findings.

In conclusion, our analyses provided convincing evidence that waist circumference might be one of the scientific and important predictor of PLC based on the large-scale prospective study. The findings indicated that both high and low waist circumference could increase the risk of PLC in males, especially for subjects with HBsAg negative. Therefore, controlling waist circumference in an appropriate range might be an effective primary prevention to decrease PLC risk.

## Data Availability

The datasets for this manuscript are not publicly available because all our data are under regulation of both the National Cancer Center of China and Kailuan Group. Requests to access the datasets should be directed to Jie He, hejie@cicams.ac.cn and Shouling Wu, drwusl@163.com.

## Author Contributions

NL, MD, SW, and JH did the study concept and design. GW, XF, YC, HC, and SC carried out the acquisition and quality control of data. LW, ZL, XL, and YW performed statistical analysis, or interpretation of data. LW performed the writing and drafting of the manuscript. NL and MD did the critical revision of the manuscript for important intellectual content. All authors agreed to be accountable for the content of the work.

### Conflict of Interest Statement

The authors declare that the research was conducted in the absence of any commercial or financial relationships that could be construed as a potential conflict of interest.

## Abbreviations

PLC: primary liver cancer
RCS: restricted cubic spline
HR: hazard ratio
CI: confidence interval
IARC: International Agency for Research on Cancer
HBV: hepatitis B virus
HBsAg: hepatitis B surface antigen
BMI: body mass index
HCV: hepatitis C virus
IGF: insulin-like growth factor.
