# Supplementary material for: Waist Circumference Might Be a Predictor of Primary Liver Cancer: A Population-Based Cohort Study
Source: Front Oncol. 2018 Dec 12;8:607. doi: 10.3389/fonc.2018.00607 (PMC6315118; doi:10.3389/fonc.2018.00607)
Supplement: Supplementary file 1 [file Table_1.DOCX]

We used the following formula to calculate population attributable fraction (PAF) for categorical exposure variables, including HBsAg status, waist circumference, smoking and drinking:

$$\mathrm{PAF}=\frac{P_{i}(HR_{i}-1)}{P_{i}(HR_{i}-1)+1}$$

where *P_i_* is the prevalence of exposure to the risk factor in the total population, and H*R_i_* is the hazard ratio for the association between exposure category *i* and primary liver cancer incidence.

| **Supplementary Table S1** Summary of population attributable risk (PAF%) estimates. | | |
| --- | --- | --- |
| **Risk factor** | *HR (*95%*CI)* | PAF% |
| **HBsAg status** |  |  |
| Negative | ref | 45.64% |
| Positive | 26.07 (21.07-32.24) |  |
| **Waist circumference** |  |  |
| < 80.0 | 1.39 (0.97 -2.00) | 23.01% |
| 80.0 ~ 84.9 | 1.10 (0.76 -1.57) |  |
| 85.0 ~ 89.9 | ref |  |
| 90.0 ~ 94.9 | 1.12 (0.78 -1.60) |  |
| ≥95.0 | 1.86 (1.36 -2.54) |  |
| **Smoking** |  |  |
| Non-smoker | ref | 7.55% |
| Ex-smoker | 1.10 (0.64-1.90) |  |
| Current smoker | 1.20 (0.96-1.50) |  |
| **Drinking** |  |  |
| Non-drinker | ref | 4.13% |
| Ex-drinker | 1.80 (1.21-2.69) |  |
| Current drinker ^a^ | 1.02 (0.78-1.33) |  |
| ^a^ Including <1 time per day and ≥1 time per day | | |
